# Impact of serious mental illness on the treatment and mortality of older patients with locoregional high‐grade (nonmetastatic) prostate cancer: retrospective cohort analysis of 49 985 SEER‐Medicare patients diagnosed between 2006 and 2013

**DOI:** 10.1002/cam4.2109

**Published:** 2019-04-03

**Authors:** Dennis A. Fried, Hossein Sadeghi‐Nejad, Dian Gu, Shouhao Zhou, Weiguo He, Sharon H. Giordano, Sri Ram Pentakota, Kitaw Demissie, Drew Helmer, Chan Shen

**Affiliations:** ^1^ War Related Illness and Injury Study Center VA‐New Jersey Healthcare System East Orange New Jersey; ^2^ Department of Epidemiology Rutgers, The State University of New Jersey Newark New Jersey; ^3^ VA New Jersey Health Care System East Orange New Jersey; ^4^ MD Andersen Cancer Center, University of Texas Houston Texas; ^5^ College of Medicine Penn State University Hershey Pennsylvania; ^6^ SUNY Downstate School of Public Health Brooklyn New York; ^7^ New Jersey Medical School, Rutgers, The State University of New Jersey Newark New Jersey

## Abstract

**Background:**

The influence of serious mental illness (SMI) on the treatment and survival of patients with high‐grade prostate cancer is not well understood. We compared the initial cancer treatment and cancer‐specific mortality of SEER‐Medicare patients with locoregional high‐grade (nonmetastatic) prostate cancer with and without preexisting SMI.

**Methods:**

We identified SEER‐Medicare patients who were 67 years of age or older diagnosed between 2006 and 2013 with locoregional high‐grade (nonmetastatic) prostate cancer. Preexisting SMI was identified by claims indicative of bipolar disorder, schizophrenia, and other psychotic disorder, during the 2 years before cancer diagnosis. We used multivariable binary logistic regression to examine associations between SMI and receipt of surgery or radiation concurrent with hormone therapy (definitive initial treatment) within 1 year after cancer diagnosis. We used Kaplan‐Meier survival curves, as well as Cox proportional hazards and competing risk models to evaluate unadjusted and adjusted associations between SMI and 5‐year cancer‐specific survival.

**Results:**

Among 49 985 patients with locoregional high‐grade (nonmetastatic) prostate cancer, 523 (1.1%) had SMI and 49 462 (98.9%) had no SMI. Overall, SMI was associated with reduced odds of receiving surgery (OR = 0.66, 95% CI: 0.49‐0.89) or radiation concurrent with hormone therapy (OR = 0.81, 95% CI: 0.67‐0.98) as initial treatments in the year after cancer diagnosis. Additionally, SMI was associated with higher hazard of 5‐year cancer‐specific death (HR = 1.41, 95% CI: 1.06‐1.89) after accounting for competing risks of non‐cancer death.

**Conclusion:**

Among SEER‐Medicare patients with locoregional high‐grade (nonmetastatic) prostate cancer, those with preexisting SMI—relative to those without these conditions—were less likely to receive definitive initial treatment in the year after diagnosis and had poorer cancer‐specific survival 5 years after diagnosis.

## 1 INTRODUCTION

Prostate cancer is one of the most prevalent cancers representing almost 10% of incident cancer cases in the US.^1^ It is the most common non‐skin cancer among American men, with black men and those with low socioeconomic status being disproportionately burdened.^2^ In 2020, it is estimated that among US adults there will be 235 000 new prostate cancer cases and 28 000 prostate cancer deaths.^3^

Mental illness is also highly prevalent among US adults, with one in five (44.7 million in 2016) living with a mental illness and one in two facing a diagnosis in their lifetime.^4^ Mental illness encompasses a spectrum of conditions that vary in severity. “Serious mental illness” (SMI)—a more severe subset of mental illness—is defined as a mental, behavioral, or emotional disorder resulting in functional impairment which substantially interferes with or limits one or more major life activities.^5^ Despite comparable cancer incidence,^6^, ^7^ individuals with SMI nevertheless have higher cancer mortality than those without SMI, after adjusting for confounding factors such as smoking status.^6^

Numerous factors beyond the biologic characteristics of prostate cancer may be associated with mortality risk. Studies suggest that treatment‐related differences contribute to higher cancer mortality among those with SMI.^7^ Patients with SMI encounter barriers to care: for instance, patients with SMI are less likely than those without SMI to receive cancer screening and/or preventive care services.^8^ Moreover, cancer patients with SMI are less likely to receive definitive treatment.^7^ While numerous studies have examined the treatment and all‐cause survival of prostate cancer patients with low or intermediate grade disease,^9^, ^10^ few if any have compared the treatment and cancer‐specific survival of high‐grade prostate cancer patients with and without SMI.

The purpose of this study was to examine initial cancer treatment and 5‐year cancer‐specific survival among a large retrospective cohort of SEER‐Medicare patients with locoregional high‐grade (nonmetastatic) prostate cancer, with vs without preexisting SMI. We hypothesized that SMI would be associated with reduced likelihood of receiving definitive initial treatment (ie, surgery or radiation concurrent with hormone therapy within 1 year from cancer diagnosis) and lower 5‐year cancer‐specific survival.

## 2 METHODS

We used Surveillance, Epidemiology, and End Results (SEER) cancer registry data linked with Medicare claims files for this retrospective analysis. The SEER cancer registry covers 18 geographic areas of the United States and approximately 28% of the United States population. As such, it serves as an important population‐based data source for cancer research. The SEER dataset includes information on patients’ demographics, tumor characteristics, and cause of death information for those who died in the US.^11^ The linked Medicare claims data provide additional information on patients’ Medicare enrollment and health care utilization. In the SEER‐Medicare linked database, records of approximately 93% of SEER patients aged 65 and older are linked with Medicare enrollment records. The SEER‐Medicare linked database has been widely used to examine treatment patterns and survival among older patients with cancers.^12^

### 2.1 Study population

We initially selected 167 228 SEER‐Medicare patients who were 67 years of age or older at time of prostate cancer diagnosis and who had been diagnosed between January 2006 and December 2013 with locoregional high‐grade (nonmetastatic) prostate cancer. “High‐grade” refers to tumors with Gleason scores of 7‐10 (aggressive tumors).^13^ We focus on patients with “high‐grade” prostate cancer because *first*, American Urological Association (AUA) clinical treatment guidelines^13^ during our study period recommended surgery or radiation concurrent with hormone therapy as initial treatments for these patients; *second*, these recommendations were largely unchanged during our study period. Importantly, selection of patients 67 years of age or older (at diagnosis) allowed us to use a 2‐year lookback period to identify those SEER‐Medicare patients who had a recorded diagnosis of SMI in the 2 years prior to their prostate cancer diagnosis.

**Figure 1:**
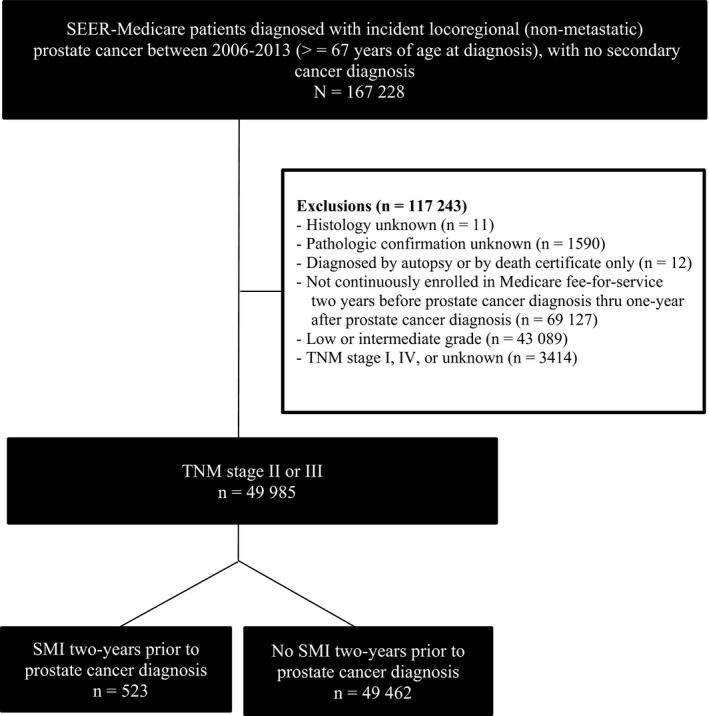
Selection of SEER‐Medicare patients diagnosed with locoregional high‐grade (nonmetastatic) prostate cancer between 2006 and 2013

To be included in the study cohort, patients had to have continuous fee‐for‐service enrollment in Medicare Parts A and B (with no health maintenance organization enrollment) in the period from 2 years before through 1 year after prostate cancer diagnosis, to ensure more complete records. As further inclusion criteria, we restricted our cohort to patients with locoregional stage, high‐grade/nonmetastatic disease to minimize the chances of selecting patients whose cancers had spread or those with low‐grade disease who may have been candidates for observation. In the process, we excluded patients with unknown histology; no pathologic confirmation; cancer diagnosis at autopsy or by death certificate only; low or intermediate grade; TNM stage I or IV or missing; tumors which had spread to the lymph nodes or metastasized elsewhere. The final cohort (Figure 1) consisted of 49 985 locoregional prostate cancer patients with high‐grade/nonmetastatic disease.

### 2.2 Outcomes

#### 2.2.1 Initial treatment

In examining initial cancer treatment within 1 year after prostate cancer diagnosis, we operationalized two separate binary (Yes/No) treatment outcomes. Consistent with AUA clinical treatment guidelines for our study period,^13^ we examined initial receipt of *surgery* (ie, radical prostatectomy) or *radiation concurrent with hormone therapy* within 1 year after prostate cancer diagnosis. In ascertaining initial treatment, patients were followed from the date of their prostate cancer diagnosis until the end of December 2014 (most‐recent year available). Initial treatment information was extracted from Medicare claims using International Classification of Diseases, Ninth Revision, Clinical Modification (ICD‐9‐CM codes), Current Procedural Terminology (CPT codes) and Healthcare Common Procedure Coding System (HCPCS codes) (Appendix S1).

#### 2.2.2 Survival

In examining 5‐year cancer‐specific survival as a separate outcome, patients were followed from the date of their prostate cancer diagnosis until the end of 2013 (most‐recent year available). Information on each patients’ vital status, survival time, and cause of death was extracted from their SEER registry record. We used this information to ascertain whether each patient died as a result of cancer or other non‐cancer cause, or was alive at the end of 2013.^14^

### 2.3 Main variable of interest

The main variable of interest was a dichotomous variable representing the presence or absence of SMI during the 2 years before prostate cancer diagnosis. ICD‐9‐CM codes reported on Medicare claims during the 2‐year period prior to prostate cancer diagnosis were reviewed to identify SMI including bipolar disorder, schizophrenia, and other psychotic disorder (296.0, 296.1, 296.4, 296.5, 296.6, 296.7, 296.8, 296.9, 295, 297, 298, 293.81, 293.82).^8^, ^14^ A prostate cancer patient was considered to have preexisting SMI if a relevant ICD‐9 code was present on at least one inpatient or two outpatient claims during the 2 years prior to prostate cancer diagnosis.^14^

Although the primary focus of this study was SMI, we also identified patients with major depressive disorder (MDD). A prostate cancer patient was considered to have preexisting MDD if a relevant ICD‐9 code (“296.2”—major depressive disorder single episode; “296.3”—major depressive disorder recurrent episode) was present on at least one inpatient or two outpatient claims during the 2 years prior to prostate cancer diagnosis.^15^

### 2.4 Other covariates

As covariates, patient characteristics and neighborhood socioeconomic status characteristics were obtained from the SEER record. We included the following demographic variables: age at time of prostate cancer diagnosis (<70, 70‐74, and ≥75 years), race/ethnicity (non‐Hispanic white, non‐Hispanic black, and Hispanic/non‐Hispanic others), region (Northeast, West, Midwest, South), and urban/rural residence status. Since being married is associated with reduced morbidity and mortality,^16^ we included marital status as a three‐level categorical variable: married, unmarried (ie, single, unmarried, separated, divorced, or widowed), and unknown/missing. We also included the following neighborhood socioeconomic status variables: neighborhood median income, percentage below poverty level, and percentage with more than a high school education, with all three measures in quartiles.

To account for differences in comorbidity burden between those with and without SMI, we computed Charlson comorbidity index score (Deyo‐Romano adaptation). The Charlson score assesses the overall burden of disease and is associated with mortality (higher scores are associated with higher mortality).^17^ The Charlson comorbidity score is a commonly adopted measure for ascertainment of comorbidity in studies using Medicare claims data.^18^, ^19^, ^20^, ^21^ The continuous Charlson comorbidity score was categorized into three groups: 0, 1, or 2+. Charlson comorbidity scores were derived from the Medicare claims records during the year prior to prostate cancer diagnosis.

Because stage at diagnosis is an important determinant of cancer outcomes,^22^ TNM stage at diagnosis was a covariate in this analysis. Tumor stage refers to the degree to which the tumor is confined to the prostate gland or has spread.^13^ Since, we restricted our cohort to patients with TNM stages II or III (to minimize the chances of selecting patients with metastases or those with low‐grade disease who may not require treatment), we operationalized TNM stage as a two‐level (II/III) adjustment variable.

### 2.5 Statistical analyses

For categorical variables, we report frequencies and percentages by SMI status, using chi‐squared to test for significant subgroup differences. For continuous variables, we report means (standard errors) and medians (interquartile ranges), with Wilcoxon rank sum or *t* tests used to detect significant subgroup differences. We conducted multivariable binary logistic regression to examine associations between SMI status and receipt of initial cancer treatments, controlling for all other covariates. Further, we conducted unadjusted survival analyses using Kaplan‐Meier estimation. We also used Cox proportional hazards modeling and competing risk modeling to evaluate adjusted associations between SMI and 5‐year cancer‐specific survival. The competing risk modeling was used to examine associations between SMI and cancer‐specific death accounting for competing risks of death from non‐cancer causes. We provide the logrank test, hazard ratios (HRs), and corresponding 95% confidence intervals (CIs) from the multivariable analyses. Finally, in multivariable modeling, missing observations (less than 5%) were deleted through listwise deletion.

#### 2.5.1 Sensitivity analysis

Major depressive disorder is a relatively common mental health condition, with almost 5% of all US adults in any given year having at least one major depressive episode.^5^ MDD can result in severe impairments that interfere with or limit one's ability to carry out major life activities.^5^ Importantly, while the National Institute of Mental Health considers MDD to be a subtype of SMI, not all studies have included MDD in their definitions of SMI.^8^, ^14^ In this study, we separately examine multivariable associations between SMI and initial cancer treatment and cause‐specific mortality using two different definitions of SMI (SMI without vs with MDD). Sensitivity analysis results are provided in Appendices S2‐S5.

The MD Anderson Cancer Center Institutional Review Board approved this study. All analyses were performed with SAS Enterprise Guide 6.1 (SAS Corp: Cary, NC). Statistical tests were two‐tailed and conducted with *α* = 0.05 significance level.

## 3 RESULTS

Among 49 985 older men who were diagnosed with incident locoregional high‐grade (nonmetastatic) prostate cancer between 2006 and 2013, approximately 523 (1.1%) of these patients had been given a documented diagnosis of SMI by a physician in the 2 years before prostate cancer diagnosis. Overall, patients with SMI were more likely than those without SMI to be older, non‐Hispanic black race (18.0% vs 10.1%, *P* < 0.0001), unmarried (39.4% vs 19.6%, *P* < 0.0001), reside in higher poverty census tracts (29.6% vs 25% were in the fourth quartile, *P* = 0.0194), or to have Charlson scores of 2+ (38.2% vs 16.5%, *P* < 0.0001). In contrast, patients with SMI were less likely than those without SMI to receive surgery (12.8% vs 21.8%, *P* < 0.0001) within 1 year after prostate cancer diagnosis (Table 1).

**Table 1:** Characteristics of SEER‐Medicare patients with locoregional high‐grade (nonmetastatic) prostate cancer, with and without serious mental illness^a^

	Serious mental illness (Yes)	Serious mental illness (No)	*P* b
N (%)	523 (1.1)	49 462 (98.9)	
Year of diagnosis (n, %)			0.3755
2006	79 (15.1)	7296 (14.8)	
2007	81 (15.5)	7469 (15.1)	
2008	65 (12.4)	6862 (13.9)	
2009	56 (10.7)	6275 (12.7)	
2010	58 (11.1)	6162 (12.5)	
2011	81 (15.5)	6122 (12.4)	
2012	54 (10.3)	4848 (9.8)	
2013	49 (9.4)	4428 (9.0)	
Age at diagnosis, years (n, %)			0.7478
67‐69	124 (23.7)	11 043 (22.3)	
70‐74	178 (34.0)	17 039 (34.5)	
≥75	221 (42.3)	22 380 (43.2)	
Charlson score (n, %)			<0.0001
0	177 (33.8)	29 684 (60.0)	
1	146 (27.9)	11 632 (23.5)	
≥2	200 (38.2)	8146 (16.5)	
Race/ethnicity (n, %)			<0.0001
Non‐Hispanic white	385 (73.6)	38 010 (76.9)	
Non‐Hispanic black	94 (18.0)	5016 (10.1)	
Hispanic/non‐Hispanic others	44 (8.4)	6436 (13.0)	
Marital status (n, %)			<0.0001
Married	245 (46.9)	33 192 (67.1)	
Unmarried	206 (39.4)	9673 (19.6)	
Unknown/missing	72 (13.8)	6597 (13.3)	
Census tract median income (n, %)			0.0384
First quartile ($20 999‐$43 741)	159 (30.4)	12 540 (25.4)	
Second quartile ($43 742‐$54 207)	138 (26.4)	12 482 (25.2)	
Third quartile ($54 208‐$64 588)	118 (22.6)	12 200 (24.7)	
Fourth quartile ($64 589‐$112 115)	108 (20.7)	12 240 (24.8)	
Census tract % below poverty level (n, %)			0.0194
First quartile (1.1%‐10.1%)	114 (21.8)	12 675 (25.6)	
Second quartile (10.2%‐12.9%)	124 (23.7)	12 638 (25.6)	
Third quartile (13%‐17.4%)	130 (24.9)	11 806 (23.9)	
Fourth quartile (17.5%‐48%)	155 (29.6)	12 343 (25.0)	
Census tract % above high school (n, %)			0.1329
First quartile (56.8%‐81.6%)	141 (27.0)	12 411 (25.1)	
Second quartile (81.7%‐86.4%)	149 (28.5)	12 522 (25.3)	
Third quartile (86.5%‐89.8%)	123 (23.5)	12 600 (25.5)	
Fourth quartile (89.9%‐99.3%)	110 (21.0)	11 929 (24.1)	
Urban/rural residence (n, %)			0.9234
Metropolitan	433 (82.8)	41 029 (83.0)	
Nonmetropolitan	90 (17.2)	8433 (17.0)	
Geographic region (n, %)			0.0164
Midwest	74 (14.2)	6585 (13.3)	
Northeast	111 (21.2)	9104 (18.4)	
South	148 (28.3)	12 411 (25.1)	
West	190 (36.3)	21 362 (43.2)	
TNM summary staging (n, %)			0.0179
Stage II	485 (92.7)	44 717 (90.4)	
Stage III	38 (7.3)	4745 (9.6)	
Received surgery (n, %)	67 (12.8)	10 775 (21.8)	<0.0001
Received radiation concurrent with hormone therapy (n, %)	156 (29.8)	16 554 (33.5)	0.0792

SMI = serious mental illness; MDD‐major depressive disorder; Data presented as percentage unless otherwise noted. SD = standard deviation.

a SMI defined as one inpatient or two outpatient ICD‐9 codes (2 years prior to diagnosis) for schizophrenia, bipolar disorder, or other psychotic disorder.

b Statistically significant difference determined by chi‐squared test test (categorical variables) or *t* test (continuous variables).

Multivariable logistic regression modeling revealed that among high‐grade prostate cancer patients, those with SMI had 34% (OR = 0.66, 95% CI: 0.49‐0.89) and 19% (OR = 0.81, 95% CI: 0.67‐0.98) lower odds of receiving surgery or radiation concurrent with hormone therapy, respectively, within 1 year from cancer diagnosis (Table 2).

**Table 2:** Multivariable binary logistic regression modeling of the associations between serious mental illness^a^ and receipt of surgery or radiation concurrent with hormone therapy among SEER‐Medicare patients with locoregional high‐grade (nonmetastatic) prostate cancer

	Serious mental illness (Yes)	
	Surgery OR (95% CI)	Radiation concurrent with hormone therapy OR (95% CI)
Serious mental illness (Ref = No)	0.66 (0.49‐0.89)	0.81 (0.67‐0.98)
Year of diagnosis (Ref = 2006)		
2007	1.20 (1.09‐1.32)	1.00 (0.93‐1.07)
2008	1.25 (1.13‐1.38)	0.90 (0.83‐0.96)
2009	1.29 (1.17‐1.43)	0.87 (0.81‐0.94)
2010	1.38 (1.25‐1.53)	0.90 (0.84‐0.97)
2011	1.48 (1.34‐1.63)	0.93 (0.86‐1.00)
2012	1.40 (1.26‐1.56)	0.98 (0.98‐1.06)
2013	1.37 (1.23‐1.52)	1.00 (0.92‐1.08)
Age at diagnosis, years (Ref = 67‐69 years)		
70‐74	0.48 (0.46‐0.51)	1.57 (1.49‐1.66)
≥75	0.08 (0.07‐0.08)	1.64 (1.55‐1.72)
Charlson score (Ref = 0)		
1	0.75 (0.71‐0.80)	1.19 (1.14‐1.25)
≥2	0.47 (0.43‐0.51)	1.12 (1.06‐1.18)
Race/ethnicity (Ref = Non‐Hispanic white)		
Non‐hispanic black	0.50 (0.45‐0.55)	0.91 (0.85‐0.97)
Hispanic/non‐hispanic others	0.75 (0.69‐0.81)	1.08 (1.01‐1.14)
Marital status (Ref = Married)		
Unmarried	0.54 (0.51‐0.58)	0.93 (0.89‐0.98)
Missing/unknown	0.34 (0.31‐0.38)	0.70 (0.66‐0.74)
Census tract median income (Ref = First quartile, $20,999‐$43,741)		
Second quartile ($43,742‐$54,207)	1.08 (0.98‐1.20)	0.90 (0.83‐0.97)
Third quartile ($54,208‐$64,588)	0.79 (0.69‐0.90)	0.83 (0.75‐0.92)
Fourth quartile ($64,589‐$112,115)	0.92 (0.79‐1.07)	0.78 (0.70‐0.88)
Census Tract % below poverty level (Ref = first quartile, 1.1%‐10.1%)		
Second quartile (10.2%‐12.9%)	1.03 (0.95‐1.12)	0.69 (0.64‐0.73)
Third quartile (13%‐17.4%)	1.11 (0.99‐1.25)	0.72 (0.66‐0.79)
Fourth quartile (17.5%‐48%)	0.80 (0.70‐0.93)	0.68 (0.61‐0.76)
Census Tract % above high school (Ref = first quartile, 56.8%‐81.6%)		
Second quartile (81.7%‐86.4%)	0.85 (0.78‐0.92)	1.09 (1.03‐1.16)
Third Quartile (86.5%‐89.8%)	0.82 (0.75‐0.89)	0.98 (0.91‐1.05)
Fourth Quartile (89.9%‐99.3%)	0.79 (0.72‐0.87)	0.89 (0.82‐0.96)
Urban/Rural STATUS (Ref = Metropolitan)		
Nonmetropolitan	0.75 (0.69‐0.82)	1.21 (1.14‐1.29)
Geographic region (Ref = West)		
Midwest	0.82 (0.75‐0.90)	1.06 (0.98‐1.13)
Northeast	0.49 (0.46‐0.54)	2.02 (1.91‐2.14)
South	0.66 (0.61‐0.71)	1.00 (0.94‐1.06)
TNM staging (ref = Stage II)		
Stage III	9.70 (8.99‐10.47)	0.79 (0.74‐0.85)

SEER‐Medicare data 2006‐2013; OR = Odds Ratio; 95% CI = 95% Confidence Interval.

a SMI defined as one inpatient or two outpatient ICD‐9 codes (2 years prior to diagnosis) for schizophrenia, bipolar disorder, or other psychotic disorder.

**Figure 2:**
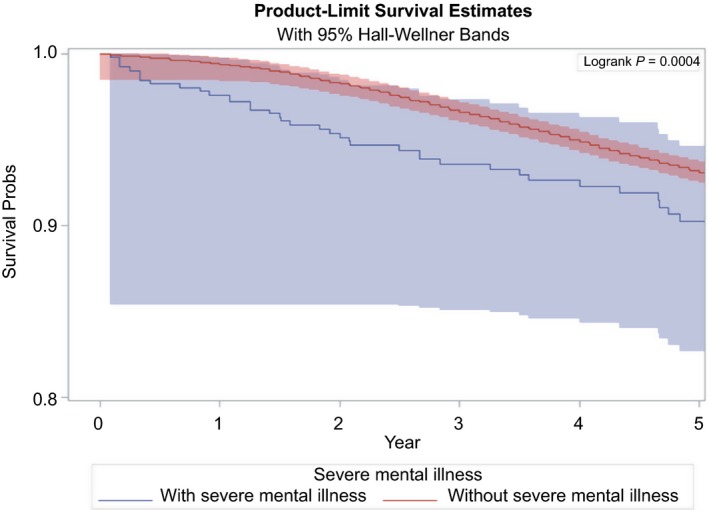
Kaplan‐Meier cancer‐specific survival curves for locoregional high‐grade (nonmetastatic) prostate cancer patients with vs without serious mental illness (SMI without major depressive disorder)

In unadjusted survival analysis, Kaplan‐Meier cancer‐specific survival curves revealed that among high‐grade prostate cancer patients, those with SMI had significantly (logrank test *P* < 0.0001) lower 5‐year cancer‐specific survival compared to those without SMI (Figure 2).

In adjusted survival analysis, multivariable Cox proportional hazards modeling revealed that among high‐grade prostate cancer patients, those with SMI had 39% (HR = 1.39, 95% CI: 1.04‐1.84) higher hazard of 5‐year cancer‐specific death. Competing risk modeling similarly revealed that patients with SMI had 41% (HR = 1.41, 95% CI: 1.06‐1.89) higher hazard of 5‐year cancer‐specific death, after accounting for competing risks of non‐cancer death (Table 3).

**Table 3:** Cox proportionate hazards and competing risk modeling of the associations between serious mental^a^ illness and cancer‐specific survival among SEER‐Medicare patients with localized, high‐grade (nonmetastatic) prostate cancer

	Serious mental illness	
	Cox proportional hazards model HR (95% CI)	Competing risk model HR (95% CI)
Serious mental illness (Ref = No)	1.39 (1.04‐1.84)	1.41 (1.06‐1.89)
Year of diagnosis (Ref = 2006)		
2007	0.98 (0.88‐1.08)	0.93 (0.84‐1.03)
2008	1.03 (0.92‐1.15)	0.94 (0.85‐1.05)
2009	1.00 (0.88‐1.14)	0.88 (0.78‐0.99)
2010	0.92 (0.79‐1.08)	0.78 (0.67‐0.90)
2011	0.91 (0.75‐1.10)	0.73 (0.61‐0.88)
2012	1.14 (0.86‐1.50)	0.84 (0.64‐1.09)
2013	0.58 (0.29‐1.19)	0.42 (0.21‐0.86)
Age at diagnosis, years (Ref = 67‐69 years)		
70‐74	1.26 (1.11‐1.43)	1.26 (1.11‐1.43)
≥75	1.99 (1.77‐2.24)	2.01 (1.79‐2.26)
Charlson score (Ref = 0)		
1	1.06 (0.97‐1.16)	1.06 (0.97‐1.16)
≥2	1.25 (1.14‐1.38)	1.26 (1.15‐1.39)
Race/ethnicity (Ref = Non‐Hispanic white)		
Non‐Hispanic black	1.02 (0.90‐1.15)	1.01 (0.90‐1.14)
Hispanic/non‐Hispanic others	0.61 (0.53‐0.70)	0.61 (0.54‐0.70)
Marital status (Ref = Not married)		
Unmarried	1.28 (1.17‐1.40)	1.29 (1.18‐1.40)
Unknown/missing	1.19 (1.07‐1.32)	1.19 (1.07‐1.32)
Census Tract median income (Ref = first quartile, $20,999‐$43,741)		
Second quartile ($43,742‐$54,207)	0.84 (0.73‐0.98)	0.84 (0.73‐0.97)
Third Quartile ($54,208‐$64,588)	0.76 (0.63‐0.91)	0.76 (0.63‐0.91)
Fourth Quartile ($64,589‐$112,115)	0.66 (0.53‐0.82)	0.66 (0.53‐0.82)
Census Tract % below poverty level (Ref = first quartile, 1.1%‐10.1%)		
Second quartile (10.2%‐12.9%)	0.94 (0.83‐1.07)	0.94 (0.83‐1.07)
Third quartile (13%‐17.4%)	0.93 (0.79‐1.10)	0.93 (0.79‐1.11)
Fourth quartile (17.5%‐48%)	0.82 (0.66‐1.01)	0.82 (0.67‐1.01)
Census Tract % above high school (Ref = first quartile, 56.8%‐81.6%)		
Second quartile (81.7%‐86.4%)	0.93 (0.83‐1.04)	0.93 (0.83‐1.04)
Third quartile (86.5%‐89.8%)	0.93 (0.82‐1.05)	0.93 (0.82‐1.06)
Fourth quartile (89.9%‐99.3%)	0.92 (0.80‐1.06)	0.93 (0.81‐1.06)
Urban/Rural status (Ref = Metropolitan)		
NonMetropolitan	0.99 (0.89‐1.12)	1.00 (0.89‐1.13)
Geographic region (Ref = West)		
Midwest	0.85 (0.74‐0.97)	0.85 (0.74‐0.97)
Northeast	0.98 (0.87‐1.10)	0.98 (0.87‐1.10)
South	0.99 (0.89‐1.10)	0.99 (0.89‐1.10)
Received surgery (ref = No)	0.34 (0.29‐0.39)	0.34 (0.29‐0.39)
Received Radiation + ADT (ref = No)	0.81 (0.75‐0.88)	0.80 (0.74‐0.87)
TNM Staging (ref = Stage II)		
Stage III	1.72 (1.49‐1.97)	1.71 (1.49‐1.96)

SEER‐Medicare data 2006‐2013; HR = Hazard Ratio; 95% CI = 95% Confidence Interval.

a SMI defined as one inpatient or two outpatient ICD‐9 codes (2 years prior to diagnosis) for schizophrenia, bipolar disorder, or other psychotic disorder.

Overall, sensitivity analyses (Appendices S2‐S5) revealed that, including MDD patients in the SMI cohort produced similar‐sized associations between SMI and treatment and mortality, after adjusting for all other covariates. These associations were not statistically significant when including MDD in the SMI cohort, however. For this reason, we present results based on SMI without MDD.

## 4 DISCUSSION

The effect of SMI on the treatment and mortality of older prostate cancer patients in the United States represents an understudied, but important area of research. In the current analysis, among a cohort of older men who were diagnosed with incident locoregional high‐grade (nonmetastatic) prostate cancer between 2006 and 2013, we compared initial cancer treatment and cancer‐specific survival for those with and without *preexisting* SMI. Overall, we found that SMI was associated with lower likelihood of receiving definitive initial cancer treatment in the year after diagnosis, as well as higher cancer‐specific mortality 5 years after diagnosis. These findings suggest that barriers to treatment of locoregional high‐grade prostate cancer may continue to exist for some patients with SMI.

To facilitate care decisions, it is worth reiterating that AUA clinical guidelines strongly recommended radical prostatectomy or radiation concurrent with hormone therapy as standard initial treatment options for patients with localized or regional high‐risk prostate cancer during our study period.^13^ In the context of this study, prostate cancer patients with preexisting SMI were less likely than those without mental illness to receive initial surgery or radiation concurrent with hormone therapy for their high‐grade cancer in the year after diagnosis, a finding consistent with prior research in people with other cancers. A study of more than 24 000 older adults with breast cancer found that women with (vs without) a prior diagnosis of depression had a lower likelihood of receiving definitive cancer treatment^23^; a study of 160 veterans found that patients with preexisting psychiatric comorbidities were less likely than those without these conditions to receive surgery for esophageal cancer^24^; an Australian study of 6586 cancer patients found that compared to the general population, patients with psychiatric conditions were less likely to receive surgery or radiation or chemotherapy for colorectal, breast, cervical, or uterine cancers^25^; an analysis of 86 670 SEER‐Medicare patients with colon cancer similarly showed that patients with preexisting mental health conditions were less likely than those without these conditions to receive surgery, chemotherapy, or radiation therapy.^26^

The finding that among our cohort, those with SMI were less likely to receive definitive initial treatment has implications for mortality risk, as lower likelihood of receiving guideline concordant care is generally associated with higher mortality.^27^ In examining 5‐year cancer‐specific mortality among high‐grade prostate cancer patients, we found that SMI was associated with higher cancer‐specific mortality, after accounting for competing risks. This finding was also consistent with prior studies. A study of SEER‐Medicare patients found that preexisting mental disorder was associated with 32% higher all‐cause mortality and 33% higher colon cancer‐specific mortality compared to no mental disorder^26^; a study of patients with oral cancer found that mental illness was associated with 58% higher all‐cause mortality compared to those without mental illness^28^; a study of cancer mortality and mental illness reported that those with mental health conditions had 29%‐52% higher cancer‐specific mortality compared to those without mental health conditions^25^; a subsequent study of cancer patients found that SMI (relative to those without SMI) was associated with 5‐year cancer‐specific mortality that was 150% (breast cancer) and 200% (colorectal cancer) higher, after adjusting for confounding^27^; more recently, while not attaining statistical significance, point estimates from a SEER‐Medicare breast cancer study suggested that preexisting SMI was associated with higher breast cancer‐specific mortality.^14^

Chronic medical comorbidities likely contribute to the higher mortality of patients with cancers.^29^ In this study, 38% of patients with severe mental illness had two or more medical comorbidities, compared with only 16% of those without mental illness. Nevertheless, the extent to which comorbidities—as opposed to other factors, such as cancer site and stage—contribute to the higher cancer mortality of those with SMI cannot be resolved by this analysis. Since comorbidities likely contribute to higher mortality by impacting cancer prevention and treatment,^29^ subsequent studies should evaluate the role of an individual comorbidity on the detection, treatment, and adherence of a cohort of prostate cancer patients (with comparable tumor type and stage).

### 4.1 Limitations

This analysis has several important limitations. First, the role of behavioral factors, such as alcohol use, substance abuse, and inadequate physical activity, could not be assessed. Second, because our cohort was restricted to subjects with fee‐for‐service Medicare coverage who were 67 years of age or older when diagnosed, our findings may not be generalizable to younger populations or those enrolled in managed care plans. Third, ascertainment of SMI was based on ICD‐9‐CM codes reported in Medicare claims files which may have resulted in misclassification. Fourth, while serial treatment modalities (eg, primary treatment with radical prostatectomy, followed by salvage external beam radiation concurrent with androgen deprivation therapy) can be part of a patients’ cancer care trajectory in the year after diagnosis, a limitation of SEER data is that it only provides information on “first‐course” of treatment. Consequently, we were unable to examine initial serial prostate cancer treatment modalities other than radiation concurrent with hormone therapy. This study also has important strengths: we used a large cohort of patients with high‐grade prostate cancer; our analytic approach accounted for the presence of competing risks; few studies have considered the impact of preexisting SMI diagnosis on the definitive initial treatment and cancer‐specific survival of older patients with locoregional high‐grade prostate cancer.

## 5 CONCLUSION

In conclusion, among older patients who were diagnosed with incident locoregional high‐grade prostate cancer between 2006 and 2013, those with SMI were less likely than those without mental illness to receive definitive initial cancer treatment and had higher cancer‐specific mortality 5 years after diagnosis. These findings suggest that SMI may be a barrier to receipt of initial definitive treatment at least for some older patients with locoregional high‐grade prostate cancer.

## 6 DISCLAIMERS

This study used the linked SEER‐Medicare database. The interpretation and reporting of these data are the sole responsibility of the authors. The authors acknowledge the efforts of the Applied Research Program, NCI; the Office of Research, Development and Information, CMS; Information Management Services (IMS), Inc; and the Surveillance, Epidemiology, and End Results (SEER) Program tumor registries in the creation of the SEER‐Medicare database.

## CONFLICT OF INTEREST

All authors declare no conflict of interest.
