# Supplementary material for: Impact of serious mental illness on the treatment and mortality of older patients with locoregional high‐grade (nonmetastatic) prostate cancer: retrospective cohort analysis of 49 985 SEER‐Medicare patients diagnosed between 2006 and 2013
Source: Cancer Med. 2019 Apr 3;8(5):2612–22. doi: 10.1002/cam4.2109 (PMC6536920; doi:10.1002/cam4.2109)
Supplement: Supplementary file 1 [file CAM4-8-2612-s001.docx]

| **INITIAL TREATMENT CATEGORIES** | **ICD-9-CM** | **CPT** | **HCPCS** |
| --- | --- | --- | --- |
| **SURGERY** |  |  |  |
| *Radical Prostatectomy* | 60, 60.3-60.5 | 55810, 55812, 55815, 55840, 55842, 55845, 55821, 55831, 55866 | X |
| **RADIATION** |  |  |  |
| *External Beam Therapy* | X | 77401-77416, 77418 | G0174 |
| *Brachytherapy* | 92.21-92.26 | x | x |
| **HORMONE THERAPY** |  |  |  |
| *Orchiectomy* | 62.4, 62.41, 62.42 | 54520-54535, 54690-54699 | X |
| *Oral/Solution* | X | 4179F | S0175, S0187, S0170 |
| *Injection* | X | X | J10150, J1051, J1950, J3315, J9165, C9216, C9430, G0356, J0128, S0165, S9560 |
| *Implant* | X | X | J9202, J9217, J9218, J9219 |

Notes: ICD-9-CM= International Classification of Diseases, Ninth Revision, Clinical Modification; CPT= Current Procedural Terminology; HCPCS= Healthcare Common Procedure Coding System

**Appendix I**. ICD-9-CM, CPT, and HCPCS codes used to ascertain treatment within one-year after prostate cancer diagnosis
