# Supplementary material for: Impact of serious mental illness on the treatment and mortality of older patients with locoregional high‐grade (nonmetastatic) prostate cancer: retrospective cohort analysis of 49 985 SEER‐Medicare patients diagnosed between 2006 and 2013
Source: Cancer Med. 2019 Apr 3;8(5):2612–22. doi: 10.1002/cam4.2109 (PMC6536920; doi:10.1002/cam4.2109)
Supplement: Supplementary file 2 [file CAM4-8-2612-s002.docx]

| **Appendix II**. Characteristics of SEER-Medicare patients with locoregional high-grade (non-metastatic) prostate cancer, with versus without serious mental illness (SMI with major depressive disorder) † | **SERIOUS MENTAL ILLNESS**  **(Yes)** | **SERIOUS MENTAL ILLNESS**  **(No)** | *p* |
| --- | --- | --- | --- |
| N (%) | 1,148 (2.3%) | 48,837 (97.7%) |  |
| **Year of diagnosis** (n, %) |  |  | 0.3036 |
| 2006 | 160 (13.9%) | 7,215 (14.8%) |  |
| 2007 | 172 (15.0%) | 7,378 (15.1%) |  |
| 2008 | 154 (13.4%) | 6,773 (13.9%) |  |
| 2009 | 124 (10.8%) | 6,207 (12.7%) |  |
| 2010 | 144 (12.5%) | 6,076 (12.4%) |  |
| 2011 | 162 (14.1%) | 6,041 (12.4%) |  |
| 2012 | 117 (10.1%) | 4,785 (9.8%) |  |
| 2013 | 115 (10.0%) | 4,362 (8.9%) |  |
| **Age at diagnosis, years** (n, %) |  |  | 0.0782 |
| 67-69 | 287 (25.0%) | 10,880 (22.3%) |  |
| 70-74 | 390 (34.0%) | 16,827 (34.5%) |  |
| >=75 | 471 (41.0%) | 21,130 (43.3%) |  |
| **Charlson score** (n, %) |  |  | <.0001 |
| 0 | 480 (41.8%) | 29,381 (60.2%) |  |
| 1 | 310 (27.0%) | 11,468 (23.5%) |  |
| >=2 | 358 (31.2%) | 7,988 (16.4%) |  |
| **Race/ethnicity** (n, %) |  |  | 0.0276 |
| Non-Hispanic white | 896 (78.1%) | 37,499 (76.8%) |  |
| Non-Hispanic black | 131 (11.4%) | 4,979 (10.2%) |  |
| Hispanic/non-Hispanic others | 121 (10.5%) | 6,359 (13.0%) |  |
| **Marital status** (n, %) |  |  | <.0001 |
| Married | 625 (54.4%) | 32,812 (67.2%) |  |
| Unmarried | 363 (31.6%) | 9,516 (19.5%) |  |
| Unknown/Missing | 160 (13.9%) | 6,509 (13.3%) |  |
| **Census tract median income** (n, %) |  |  | 0.5252 |
| First quartile ($20,999-$43,741) | 285 (24.8%) | 12,414 (25.4%) |  |
| Second quartile ($43,742-$54,207) | 299 (26.1%) | 1,2321 (25.2%) |  |
| Third quartile ($54,208-$64,588) | 266 (23.2%) | 12,052 (24.7%) |  |
| Fourth quartile ($64,589-$112,115) | 298 (26.0%) | 12,050 (24.7%) |  |
| **Census Tract % below poverty level** |  |  | 0.5285 |
| First quartile (1.1-10.1%) | 284 (24.7%) | 12,505 (25.6%) |  |
| Second quartile (10.2-12.9%) | 285 (24.8%) | 12,477 (25.6%) |  |
| Third quartile (13-17.4%) | 295 (25.7%) | 11,641 (23.8%) |  |
| Fourth quartile (17.5-48%) | 284 (24.7%) | 12,214 (25.0%) |  |
| **Census tract % above high school** (n, %) |  |  | 0.1138 |
| First quartile (56.8-81.6%) | 306 (26.7%) | 12,246 (25.1%) |  |
| Second quartile (81.7-86.4%) | 314 (27.4%) | 12,357 (25.3%) |  |
| Third quartile (86.5-89.8%) | 271 (23.6%) | 12,452 (25.5%) |  |
| Fourth quartile (89.9-99.3%) | 257 (22.4%) | 11,782 (24.1%) |  |
| **Urban/rural residence** (n, %) |  |  | 0.0016 |
| Metropolitan | 992 (86.4%) | 40,470 (82.9%) |  |
| Non-Metropolitan | 156 (13.6%) | 8,367 (17.1%) |  |
| **Geographic region** (n, %) |  |  | 0.2739 |
| Midwest | 145 (12.6%) | 6,514 (13.3%) |  |
| Northeast | 233 (20.3%) | 8,982 (18.4%) |  |
| South | 270 (23.5%) | 12,289 (25.2%) |  |
| West | 500 (43.6%) | 21,052 (43.1%) |  |
| **TNM summary staging** (n, %) |  |  | 0.1317 |
| Stage II | 1,053 (91.7%) | 44,149 (90.4%) |  |
| Stage III | 95 (8.3%) | 4,688 (9.6%) |  |
| **Received surgery** (n, %) | 203 (17.6%) | 10,639 (21.7%) | 0.0009 |
| **Received radiation concurrent with hormone therapy** (n, %) | 364 (31.7%) | 16,346 (33.5%) | 0.2107 |

Notes: SMI=serious mental illness; MDD-major depressive disorder; Data presented as percentage unless otherwise noted. SD=standard deviation; §Statistically significant difference determined by χ2 test (categorical variables) or t-test (continuous variables); † SMI defined as 1 inpatient or 2 outpatient ICD-9 codes (2-years prior to diagnosis) for schizophrenia, bipolar disorder, or other psychotic disorder
