# Supplementary material for: Impact of serious mental illness on the treatment and mortality of older patients with locoregional high‐grade (nonmetastatic) prostate cancer: retrospective cohort analysis of 49 985 SEER‐Medicare patients diagnosed between 2006 and 2013
Source: Cancer Med. 2019 Apr 3;8(5):2612–22. doi: 10.1002/cam4.2109 (PMC6536920; doi:10.1002/cam4.2109)
Supplement: Supplementary file 3 [file CAM4-8-2612-s003.docx]

| **Appendix III**. Multivariable binary logistic regression modeling of the associations between serious mental illness (SMI with major depressive disorder) and receipt of surgery or radiation concurrent with hormone therapy among SEER-Medicare patients with locoregional high-grade (non-metastatic) prostate cancer | **SERIOUS MENTAL ILLNESS**  **(Yes)** | **SERIOUS MENTAL ILLNESS**  **(Yes)** |
| --- | --- | --- |
|  | Surgery  OR (95% CI) | Radiation concurrent with hormone therapy  OR (95% CI) |
| **Severe Mental Illness** (Ref=No) | 0.82 (0.69-0.99) | 0.91 (0.80-1.04) |
| **Year of diagnosis** (Ref=2006) |  |  |
| 2007 | 1.20 (1.09-1.32) | 1.00 (0.93-1.07) |
| 2008 | 1.25 (1.13-1.38) | 0.90 (0.84-0.96) |
| 2009 | 1.29 (1.17-1.43) | 0.87 (0.81-0.94) |
| 2010 | 1.38 (1.25-1.53) | 0.90 (0.84-0.97) |
| 2011 | 1.48 (1.34-1.63) | 0.93 (0.86-1.00) |
| 2012 | 1.40 (1.26-1.56) | 0.98 (0.91-1.06) |
| 2013 | 1.37 (1.23-1.53) | 1.00 (0.92-1.08) |
| **Age at diagnosis, years** (Ref=67-69 years) |  |  |
| 70-74 | 0.48 (0.46-0.51) | 1.57 (1.49-1.66) |
| >=75 | 0.08 (0.07-0.08) | 1.64 (1.55-1.72) |
| **Charlson score** (Ref=0) |  |  |
| 1 | 0.75 (0.71-0.80) | 1.19 (1.14-1.25) |
| >=2 | 0.47 (0.43-0.51) | 1.12 (1.06-1.18) |
| **Race/ethnicity** (Ref=Non-Hispanic white) |  |  |
| Non-Hispanic black | 0.50 (0.45-0.55) | 0.91 (0.85-0.97) |
| Hispanic/non-Hispanic others | 0.75 (0.69-0.81) | 1.08 (1.01-1.14) |
| **Marital status** (Ref=Married) |  |  |
| Unmarried | 0.54 (0.50-0.58) | 0.93 (0.89-0.98) |
| Missing/Unknown | 0.34 (0.31-0.38) | 0.70 (0.66-0.74) |
| **Census tract median income** (Ref=First quartile, $20,999-$43,741) |  |  |
| Second quartile ($43,742-$54,207) | 1.08 (0.98-1.20) | 0.90 (0.83-0.97) |
| Third quartile ($54,208-$64,588) | 0.79 (0.69-0.90) | 0.83 (0.75-0.92) |
| Fourth quartile ($64,589-$112,115) | 0.92 (0.79-1.07) | 0.78 (0.70-0.88) |
| **Census Tract % below poverty level** (Ref=first quartile, 1.1-10.1%) |  |  |
| Second quartile (10.2-12.9%) | 1.03 (0.95-1.12) | 0.69 (0.64-0.73) |
| Third quartile (13-17.4%) | 1.12 (0.99-1.26) | 0.72 (0.66-0.79) |
| Fourth quartile (17.5-48%) | 0.80 (0.70-0.93) | 0.68 (0.61-0.76) |
| **Census Tract % above high school** (Ref=first quartile, 56.8-81.6) |  |  |
| Second quartile (81.7-86.4%) | 0.85 (0.78-0.92) | 1.09 (1.03-1.16) |
| Third Quartile (86.5-89.8%) | 0.82 (0.75-0.89) | 0.98 (0.91-1.05) |
| Fourth Quartile (89.9-99.3%) | 0.79 (0.72-0.87) | 0.89 (0.82-0.96) |
| **Urban/Rural status** (Ref=Metropolitan) |  |  |
| Non-Metropolitan | 0.75 (0.69-0.82) | 1.21 (1.14-1.29) |
| **Geographic region (Ref=West)** |  |  |
| Midwest | 0.82 (0.74-0.90) | 1.06 (0.98-1.13) |
| Northeast | 0.49 (0.45-0.54) | 2.02 (1.91-2.14) |
| South | 0.66 (0.61-0.71) | 1.00 (0.94-1.06) |
| **TNM Staging** (ref=Stage II) |  |  |
| Stage III | 9.70 (8.99-10.46) | 0.79 (0.74-0.85) |

Notes: SMI=serious mental illness; MDD-major depressive disorder; SEER-Medicare data 2006-2013; OR = Odds Ratio; 95% CI = 95% Confidence Interval
