# Supplementary material for: Impact of serious mental illness on the treatment and mortality of older patients with locoregional high‐grade (nonmetastatic) prostate cancer: retrospective cohort analysis of 49 985 SEER‐Medicare patients diagnosed between 2006 and 2013
Source: Cancer Med. 2019 Apr 3;8(5):2612–22. doi: 10.1002/cam4.2109 (PMC6536920; doi:10.1002/cam4.2109)
Supplement: Supplementary file 4 [file CAM4-8-2612-s004.docx]

| **Appendix IV**. Cox Proportionate Hazards and Competing Risk Modeling of the associations between serious mental illness (SMI with major depressive disorder) and cancer-specific survival among SEER-Medicare patients with localized, high-grade (non-metastatic) Prostate cancer | **SERIOUS MENTAL ILLNESS**  **(Yes)** | **SERIOUS MENTAL ILLNESS**  **(Yes)** |
| --- | --- | --- |
|  | Cox Proportional Hazards Model  HR (95% CI) | Competing Risk Model  HR (95% CI) |
| **Severe Mental Illness** (Ref=No) | 1.17 (0.94-1.46) | 1.19 (0.95-1.48) |
| **Year of diagnosis** (Ref=2006) |  |  |
| 2007 | 0.98 (0.88-1.08) | 0.93 (0.84-1.03) |
| 2008 | 1.03 (0.92-1.15) | 0.94 (0.84-1.05) |
| 2009 | 1.00 (0.88-1.13) | 0.88 (0.78-0.99) |
| 2010 | 0.92 (0.79-1.07) | 0.78 (0.67-0.90) |
| 2011 | 0.91 (0.75-1.10) | 0.73 (0.61-0.88) |
| 2012 | 1.14 (0.86-1.50) | 0.84 (0.64-1.09) |
| 2013 | 0.58 (0.29-1.19) | 0.43 (0.21-0.86) |
| **Age at diagnosis, years** (Ref=67-69 years) |  |  |
| 70-74 | 1.26 (1.11-1.43) | 1.26 (1.11-1.43) |
| >=75 | 1.99 (1.77-2.24) | 2.01 (1.79-2.26) |
| **Charlson score** (Ref=0) |  |  |
| 1 | 1.06 (0.97-1.16) | 1.07 (0.98-1.16) |
| >=2 | 1.26 (1.14-1.38) | 1.26 (1.15-1.39) |
| **Race/ethnicity** (Ref=Non-Hispanic white) |  |  |
| Non-Hispanic black | 1.02 (0.90-1.15) | 1.01 (0.90-1.14) |
| Hispanic/non-Hispanic others | 0.61 (0.53-0.70) | 0.61 (0.54-0.70) |
| **Marital status** (Ref=Not married) |  |  |
| Unmarried | 1.28 (1.17-1.40) | 1.29 (1.18-1.41) |
| Unknown/Missing | 1.19 (1.07-1.32) | 1.19 (1.07-1.32) |
| **Census Tract median income** (Ref=first quartile, $20,999-$43,741) |  |  |
| Second quartile ($43,742-$54,207) | 0.85 (0.73-0.98) | 0.84 (0.73-0.98) |
| Third Quartile ($54,208-$64,588) | 0.76 (0.63-0.91) | 0.76 (0.63-0.91) |
| Fourth Quartile ($64,589-$112,115) | 0.66 (0.53-0.82) | 0.66 (0.53-0.82) |
| **Census Tract % below poverty level** (Ref=first quartile, 1.1-10.1%) |  |  |
| Second quartile (10.2-12.9%) | 0.94 (0.83-1.07) | 0.94 (0.83-1.07) |
| Third quartile (13-17.4%) | 0.93 (0.79-1.10) | 0.93 (0.79-1.10) |
| Fourth quartile (17.5-48%) | 0.82 (0.66-1.01) | 0.82 (0.67-1.01) |
| **Census Tract % above high school** (Ref=first quartile, 56.8-81.6% |  |  |
| Second quartile (81.7-86.4%) | 0.93 (0.83-1.04) | 0.93 (0.83-1.04) |
| Third quartile (86.5-89.8%) | 0.93 (0.82-1.05) | 0.93 (0.82-1.06) |
| Fourth quartile (89.9-99.3%) | 0.92 (0.80-1.06) | 0.93 (0.81-1.06) |
| **Urban/Rural status** (Ref=Metropolitan) |  |  |
| Non-Metropolitan | 1.00 (0.89-1.12) | 1.00 (0.89-1.13) |
| **Geographic region** (Ref=West) |  |  |
| Midwest | 0.85 (0.74-0.97) | 0.85 (0.74-0.97) |
| Northeast | 0.98 (0.87-1.10) | 0.98 (0.87-1.10) |
| South | 0.99 (0.89-1.10) | 0.99 (0.89-1.10) |
| **Received surgery** (ref=No) | 0.34 (0.29-0.39) | 0.34 (0.29-0.39) |
| **Received Radiation+ADT** (ref=No) | 0.81 (0.74-0.87) | 0.80 (0.74-0.87) |
| **TNM Staging** (ref=Stage II) |  |  |
| Stage III | 1.72 (1.49-1.97) | 1.71 (1.49-1.96) |

Notes: SMI=serious mental illness; MDD-major depressive disorder; SEER-Medicare data 2006-2013; HR = Hazard Ratio; 95% CI = 95% Confidence Interval
