# Supplementary material for: Impact of serious mental illness on the treatment and mortality of older patients with locoregional high‐grade (nonmetastatic) prostate cancer: retrospective cohort analysis of 49 985 SEER‐Medicare patients diagnosed between 2006 and 2013
Source: Cancer Med. 2019 Apr 3;8(5):2612–22. doi: 10.1002/cam4.2109 (PMC6536920; doi:10.1002/cam4.2109)

**Appendix V**. Kaplan-Meier cancer-specific survival curves for SEER-Medicare locoregional high-grade (non-metastatic) prostate cancer patient with versus without serious mental illness (SMI with major depressive disorder)


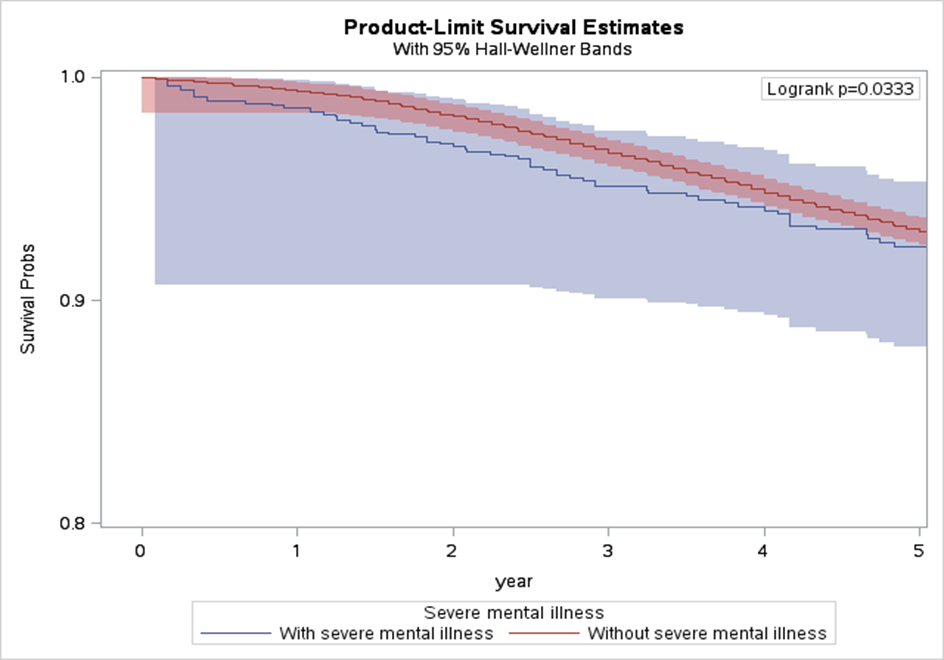

Supplement: Supplementary file 5 [file CAM4-8-2612-s005.docx]
